# Quality of life after severe acute pancreatitis: systematic review

**DOI:** 10.1093/bjsopen/zrad067

**Published:** 2023-08-24

**Authors:** Andrea Giorga, Michael Hughes, Simon Parker, Andrew Smith, Alistair Young

**Affiliations:** Department of Pancreatic Surgery, St James's University Hospital, Leeds Teaching Hospitals Trust, Leeds, UK; Department of Pancreatic Surgery, St James's University Hospital, Leeds Teaching Hospitals Trust, Leeds, UK; Organisational Behaviour and Human Resource Management, Nottingham University Business School, UK; Department of Pancreatic Surgery, St James's University Hospital, Leeds Teaching Hospitals Trust, Leeds, UK; Department of Pancreatic Surgery, St James's University Hospital, Leeds Teaching Hospitals Trust, Leeds, UK

## Abstract

**Background:**

Severe acute pancreatitis, the most severe form of acute pancreatitis, can alter pancreatic morphology, physiology, and function resulting in long-term morbidity, even after a single episode. This review assesses long-term outcomes and quality of life of severe acute pancreatitis.

**Methods:**

A comprehensive literature review was conducted across MEDLINE, Embase, Scopus, and PubMed electronic databases on 18 January 2021 and updated on 26 April 2022 to ensure no new literature had been omitted. All studies were prospective or retrospective, included adult patients (>18 years) presenting with acute pancreatitis for whom data on long-term outcomes specifically after severe acute pancreatitis were reported. Quantitative and qualitative data extraction and synthesis were carried out and no meta-analysis was performed. Outcome measures included aetiology and mortality of severe acute pancreatitis, length of stay, endocrine and exocrine pancreatic insufficiency, chronic symptoms, and quality of life compared with healthy controls as assessed by validated questionnaires.

**Results:**

Fourteen retrospective cohort studies were included, for a total of 779 patients, using quality of life questionnaires. The most common aetiology of severe acute pancreatitis was biliary (36 per cent) followed by alcoholic (29 per cent). Mortality rate ranged from 5 to 35 per cent and length of stay ranged from 2 to 367 days. Quality of life was somewhat lower in patients with exocrine insufficiency, but unaffected by endocrine insufficiency or chronic symptoms. Quality of life was more likely to be reduced in the first 4 years but normalize thereafter and was more likely to be negatively affected where alcohol was the aetiology. In four studies, the relationship between disease severity and lower quality of life was investigated, and a significant correlation was found.

**Conclusion:**

The review shows how a single episode of severe acute pancreatitis can have a variable effect on long-term quality of life, which is different to previous studies showing a strong reduction in quality of life. This could indicate that in current times treatment modalities are more effective.

## Teaser

A systematic review of studies investigating the long-term effects of severe acute pancreatitis on patients’ quality of life.

## Introduction

Acute pancreatitis (AP) refers to pancreatic inflammation with subsequent enzyme autodigestion and has an incidence of 150–420 cases per million population in the UK, with an increasing trend over the last years^1,2^. Severe acute pancreatitis (SAP) as defined by the Revised Atlanta criteria involves the presence of organ failure for over 48 hours^3^. SAP makes up 20–30 per cent of all acute cases and is associated with a high mortality rate, of up to 25 per cent^2,4–6^.

The sequelae of SAP, including pancreatic necrosis, pseudocyst, superimposed infection, and abscess, impact short-term as well as long-term patient outcomes^7,8^. Recent research on the pathophysiology of pancreatitis suggests that unlike in mild AP, the pancreas gland does not fully recover after an episode of SAP^5,9^ as it can potentially change pancreatic morphology, affecting its function to an extent which can impact patient quality of life (QoL)^10^.

Short-term outcomes resulting from early inflammatory processes have been researched extensively and can range from organ failure to death^1,7,11^, while long-term outcomes can include chronic exocrine and endocrine dysfunction^8,10^. It is also established that the aetiology of SAP affects the extent of chronic endocrine and exocrine dysfunction, with alcohol being the most detrimental cause^9^. The way in which these physiological long-term changes after SAP affect a patient’s QoL are yet to be described in a standardized way across different studies.

Traditionally, the measured outcome of AP has been mortality. As the survival rate after SAP has been increasing over time, it is important to see how SAP survivors live and how one episode changes their day-to-day activities. QoL is a widespread outcome measure in many areas of medicine and surgery and can reflect the efficacy of treatment modalities^12,13^. This study aims to review and summarize the literature from the last 30 years, assess the impact of SAP management on patient QoL, and identify any specific components that have a direct or indirect effect on QoL.

## Methods

This systematic review was carried out and reported according to PRISMA guidelines^14^. This study was registered with PROSPERO (the international prospective register of systematic reviews) on 15 January 2021 (ID: CRD42021226196). The PROSPERO database was also used to identify any completed or ongoing systematic reviews on the subject. A comprehensive literature review was conducted by a single author on 18 January 2021 and updated on 26 April 2022 to ensure no new literature had been omitted.

MEDLINE, Embase, Scopus and PubMed electronic databases were searched using medical subject heading (MeSH) terms. Google Scholar was used to search broad, non-MeSH terms. Reference lists of included studies were searched for identification of additional eligible studies. Manuscripts were screened by title and abstract by a single reviewer and subsequent full-text review was conducted by two reviewers. The literature search strategy is outlined in *Appendix S1*.

Inclusion criteria: prospective or retrospective studies, adult patients (>18 years) with acute pancreatitis, studies including severe pancreatitis cases, studies reporting the assessment of pancreatitis severity and QoL, and studies in English. Only studies reporting data separately on SAP have been included in this review. Studies involving recurrent episodes of pancreatitis were excluded.

Data was extracted using quantitative and qualitative data synthesis. Due to the limited number of included manuscripts and variability of study design and scales used, a meta-analysis was not performed.

Studies were individually assessed for bias according to study type by two reviewers, with a third assessor resolving any scoring discrepancies. The risk-of-bias assessment tool used was the Newcastle–Ottawa Scale for Cohort Studies. The scale consists of three components (Selection, Comparability, and Outcome) and a maximum total score of nine can be assigned to each study, with higher scores indicating better methodological quality. The ‘Newcastle–Ottawa' Scale was used for continuity, as was used by a systematic review on the same subject in 2015 by Pendhakhar *et al.*^12^, and has proven validity for assessment of non-randomized cohort studies^15^.

Principal summary measures included aetiology and mortality of SAP, duration of hospital stay and intensive care unit (ICU) stay, endocrine and exocrine pancreatic insufficiency, reported chronic symptoms, and a statistically significant change in at least one domain used to measure QoL compared with healthy controls.

## Results

**Fig. 1:**
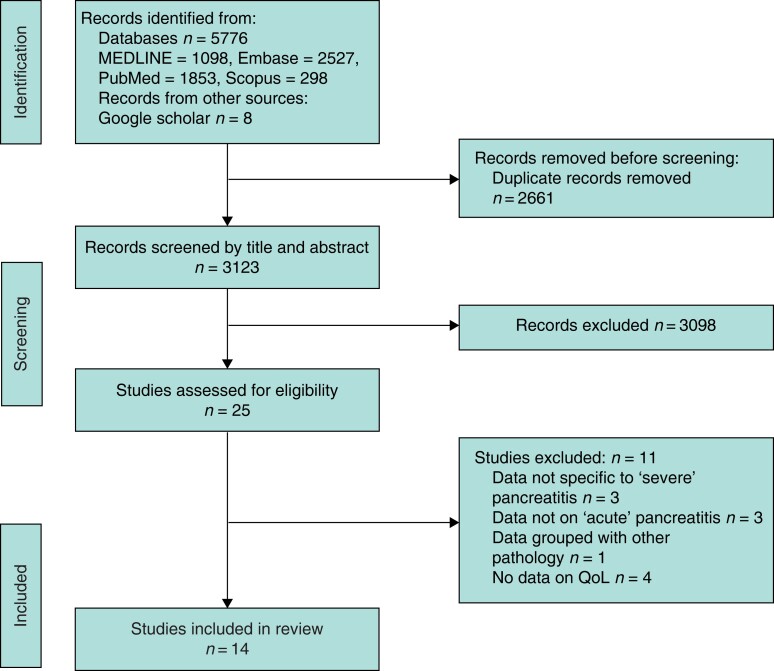
PRISMA flow diagram—overview of search strategy and identification of eligible manuscripts

Fourteen manuscripts were included in the final data synthesis, all of which were cohort studies and had carried out a formal QoL assessment using validated questionnaires (*Fig. 1*). All these studies identified patients retrospectively, and then assessed QoL in a prospective manner. Results of risk-of-bias assessment are displayed in *Table 1*.

QoL, quality of life.

**Table 1:** Newcastle–Ottawa score for risk-of-bias assessment of included studies

Study ID	Selection (max 4)	Comparability (max 2)	Outcome (max 3)	Overall score	AHRQ standards quality
Andersson^13^	2	1	2	5	Fair
Cinquepalmi^16^	3	1	2	6	Good
Gasparoto^5^	4	1	2	7	Good
Halonen^17^	3	1	2	6	Good
Hochman^18^	3	1	2	6	Good
Koziel^19^	4	1	2	7	Good
Machicado^20^	4	2	1	7	Good
Reszetow^6^	3	1	2	6	Good
Soran^21^	2	1	1	4	Poor
Symersky^22^	4	1	2	7	Good
Szentkereszty^23^	2	1	3	6	Fair
Tu^24^	3	2	2	7	Good
Wright^25^	4	2	3	9	Good
Yang^26^	4	2	2	8	Good

AHRQ, Agency for Health Research and Quality.

Eight studies were European, three North American, two Asian, and one South American. The tools used for severity assessment of pancreatitis included the Atlanta classification^3^, Ranson Criteria^27^, APACHE (Acute Physiology and Chronic Health Evaluation) II score^28^, Balthazar scoring^29^, and radiological or histological evidence of necrosis. Eleven studies investigated patients with severe pancreatitis only, while three also included mild pancreatitis patients (*Table 2*).

**Table 2:** Study characteristics, aetiology of SAP of included studies

Study ID	Setting	Patients included	Patient age & gender (M:F)	Severity assessment & score	Aetiology	Mortality after SAP	Follow-up (in months)	LOS (days)
Andersson^13^	Sweden	14/40	58 (45–67) 16:24	Atlanta	Alcohol 5/14 Biliary 4/14 Post-ERCP 3/14 Unknown 2/14	2/40	47 (37–63)	18 (16–24)
Cinquepalmi^16^	Italy	32/35	55 (44–66) 25:10	Ranson, APACHE II Radiological evidence of necrosis	Alcohol 7/35 Biliary 20/35 Iatrogenic 5/35 Idiopathic 3/35	N/A	85 (48–122)	71 (13–146)
Gasparoto^5^	Brazil	16/49	48 (35–61) 9:7	APACHE II Balthazar-Ranson Radiological evidence of necrosis	Alcohol 4/16 Biliary 10/16 Lipid disorder 2/16	11/49	35 (12–90)	21 (2–41)
Halonen^17^	Finland	145/283	44 (20–78) 120:25F	Atlanta	Alcohol 113/145 Other 32/145	99/283	19–127	39 (10–212)
Hochman^18^	Canada	25/42	59 (37–86) 16:9	Ranson	Alcohol 4/25 Biliary 11/25 Lipid disorder 2/25 Idiopathic 8/25	8/42	24–36	N/A
Koziel^19^	Poland	99/150	52 (35–69) 67:32	Atlanta	Alcohol 36/99 Biliary 42/99 Other 3/99 Idiopathic 18/99	N/A	13	N/A
Machicado^20^	USA	15/91	52 (36–69) 42:49	Atlanta	Alcohol 4/91 Biliary 39/91 Lipid disorder 9/91 Other 39/91	N/A	14	7 (4–13)
Reszetow^6^	Poland	28/30	48 (37–67) 20:8	APACHE II—Radiological evidence of necrosis	Alcohol 18/28 Biliary 10/28	9/44	61 (28–102)	*
Soran^21^	USA	39/52	53 (22–89) 22:17	APACHE II	Alcohol 6/39 Biliary 19/39 ERCP 5/39 Others 9/39	13/39	17–69	40 (6–74)
Symersky^22^	Netherlands	12/34	53 (17–78) 16:18	Atlanta	Biliary 26/34* ERCP 8/34*	N/A	12–90	N/A
Szentkereszty^23^	Hungary	22/25	46 (30–64) 19:6	Radiological and/or histological evidence of necrosis	Alcohol 18/27* Biliary 5/27* ERCP 1/27* Unknown 3/27*	N/A	37.8	N/A
Tu^24^	China	101/101	46 (42–56) 66:35	CT Severity Index APACHE II	Alcohol 37/101† Biliary 69/101† Lipid disorder 23/101†	6/109	1–192	§
Wright^25^	UK	17/31	65 (21–78) 11:6	Atlanta CT Balthazar	Alcohol 4/17 Biliary 10/17 ERCP 1/17 Idiopathic 1/17 Trauma 1/17	10/31	3, 6, & 12	82 (28–367)
Yang^26^	China	214/214	45 (35–52) 141:73	Atlanta APACHE II	Alcohol 8/214 Biliary 85/214 Lipid disorder 81/214 Other 40/214	44/214	16.7	¶

M, male; F, female; N/A, not available; APACHE II, Acute Physiology and Chronic Health Evaluation II; ERCP, Endoscopic Retrograde Cholangiopancreatography; SAP, severe acute pancreatitis; LOS, length of stay. *Aetiology for entire cohort, no values available for SAP patients only; † overlapping aetiologies; § ICU (intensive care unit) stay: ON (open necrosectomy) 20.28 ± 3.32, MID (minimally invasive drainage) 10.36 ± 2.21; ¶ ICU median stay: PICS (persistent inflammation-immunosuppression and catabolism syndrome) 42 (24–67), non-PICS 17 (16–28).

Some 779 patients with SAP were evaluated. The majority of them, 76 per cent (*n* = 590), were male and patient age ranged between 17 and 89 years. Cohort size ranged from 12 to 214 patients, with 10 studies having cohort sizes smaller than 50 and the remaining four studies having over 100 patients. Follow-up ranged from 1 to 192 months and all studies apart from two followed patients for more than 12 months. Mortality rate from the day of admission to the end of the follow-up period ranged from 5 per cent to 35 per cent.

The most commonly encountered QoL tool was the Short-Form 36 Questionnaire (SF-36), used by 11 studies^30,31^. The SF-12, a shorter version, adapted from the SF-36 in order to reduce respondent burden, was used in one study^32^. The Functional Assessment of Chronic Illness Therapy (FACIT) Measurement System, a non-disease-specific questionnaire, was used in one study^31,33^. The Gastro-Intestinal QoL Index (GIQLI), another non-disease-specific system, was used by one study^31,34^.

### Quality of life outcomes

Quality of life scores obtained from questionnaires of patients after an episode of SAP were compared with age-matched healthy controls in 10 studies (*Table 3*). Six studies demonstrated a statistically significant reduction in at least one domain of QoL compared with control^5,18,20,22,25,26^. Five studies showed no difference in the QoL of SAP patients when compared with control after at least 19 months of follow-up^6,13,17,21,23^.

**Table 3:** QoL and long-term outcomes of included studies

Study ID	Exocrine insufficiency	Endocrine insufficiency	Chronic symptoms	QoL tool	Effect on QoL
Andersson^13^	4/14	11/14 IGT and/or DM of GTT	12/14 abdominal pain 4/14 diarrhoea	SF-36	None compared with control
Cinquepalmi^16^	Nil	10/35	N/A	SF-36	None—no control Lower scores in alcohol-induced SAP
Gasparoto^5^	1/16	12/16	N/A	SF-36	Significantly reduced mental health compared with control (*P* = 0.028)
Halonen^17^	11/145	68/145	91/145 abdominal pain	SF-36	None compared with control
Hochman^18^	16/25	8/25	11/25 abdominal pain	SF-36	Significantly reduced physical health compared with control (*P* < 0.001)
Koziel^19^	17/99	16/99	N/A	SF-36	Significantly reduced mental health (*P* < 0.050)—no healthy control
Machicado^20^	N/A	5/91	21/91 abdominal pain 6/91 disability	SF-12	Significantly reduced physical health (*P* < 0.050) compared with control
Reszetow^6^	4/28	22/28	N/A	FACIT	None compared with control Alcohol lower QoL
Soran^21^	N/A	N/A	N/A	SF-36	None compared with control
Symersky^22^	9/12	5/12	N/A	GIQLI	Significantly reduced QoL compared with control (*P* = 0.024)
Szentkereszty^23^	7/22	3/22	9/22 abdominal distention 13/22 nausea & vomiting	SF-36	None compared with control
Tu^24^	65/101	25/101	9/101 abdominal pain 11/101 abdominal distention	SF-36	ON group significantly lower QoL than MID group (*P* < 0.020)—no comparison to healthy control
Wright^25^	N/A	N/A	N/A	SF-36	Significantly reduced compared with control at 12 months (*P* < 0.020)
Yang^26^	N/A	N/A	N/A	SF-36	Significantly reduced compared with control in 6 of 8 domains (*P* < 0.001)*

SF-36, Short Form 36 questionnaire; N/A, not available; SAP, severe acute pancreatitis; FACIT, Functional Assessment of Chronic Illness Therapy (FACIT) Measurement System; GIQLI, Gastro-Intestinal QoL Index; IGT, impaired glucose tolerance; DM, diabetes mellitus; GTT, glucose tolerance test; ON, open necrosectomy; MID, minimally invasive drainage; Exocrine insufficiency is in the form of steatorrhea or diarrhoea, reduced faecal elastase, or requirement of enzyme supplements in the long run. *Significantly reduced (*P* < 0.001) in 6 of 8 domains compared with control apart from ‘Bodily pain’ and ‘Role Emotional’.

Three studies did not compare QoL scores to healthy controls^16,19,24^. In one study, most patients (>68 per cent) scored the equivalent of a ‘Good/Fair’ QoL^16^. An additional study reported a statistically significant reduction in the mental health component of QoL compared to patients with mild AP^19^.

Eight studies used the SF-36 as a QoL tool, with comparison to age-matched controls, and provided a detailed analysis of the results for each domain of the questionnaire^5,13,18,21,22,25,26,32^ (*Table 4*).

**Table 4:** Individual SF-36 domains compared with controls from studies using this scale to assess QoL

	Andersson^13^	Gasparoto^5^	Halonen^17^	Hochman^18^	Koziel^19^	Soran^21^	Szentkereszty^23^	Wright^25^
Physical function	Same	Same	Same	Reduced	Same	Reduced	Reduced	Reduced
Physical role	Same	Same	Same	Reduced	Same	Reduced	Reduced	Reduced
Bodily pain	Same	Same	Same	Same	Same	Same	Reduced	Reduced
General health	Same	Same	Reduced	Reduced	Same	Reduced	Reduced	Reduced
Energy/vitality	Same	Same	Same	Same	Same	Reduced	Reduced	Same
Social functioning	Same	Same	Same	Reduced	Reduced	Same	Same	Reduced
Emotional role	Reduced	Same	Same	Reduced	Reduced	Reduced	Reduced	Reduced
Mental health	Same	Reduced	Same	Same	Reduced	Same	Reduced	Reduced
**Overall**	**Same**	**Same**	**Same**	**Reduced**	**Same**	**Reduced**	**Reduced**	**Reduced**

29 ‘Same’ domains indicating no change in QoL *versus* 27 ‘Reduced’ suggesting a lower QoL. QoL, quality of life.

### Quality of life and time

A comparison between time after an episode of SAP and QoL was made by only one study, indicating that QoL scores, and in particular the Physical Function and Physical Component Summary, were reduced mainly at the 3-month mark^25^. QoL was rated slightly higher at 6 months post-SAP and even higher at 12 months. However, all scores were significantly lower compared with a healthy control.

An indirect comparison was made by plotting the time point at which QoL was assessed and the reported QoL outcome. Despite the large error margins, studies reporting a reduced QoL were those with the shortest follow-up times (<51 months), while QoL was reported to be unchanged by all studies following up patients for a longer time (>51 months) except one^19^ (*Fig. 3*).

### Quality of life and disease severity

The relationship between disease severity and lower QoL was directly investigated by four studies. The first showed a significant correlation between higher Ranson scores and lower QoL, particularly in the physical component^18^. The second indicated a statistically significant correlation between patients who had multiple system organ failure secondary to AP and a lower QoL compared with those who did not^20^. Another study reported a reduction in the mental aspect of QoL in patients with the more severe form of the disease compared with mild pancreatitis patients^19^. Another study found that patients who developed Persistent Inflammation-Immunosuppresion and Catabolism Syndrome (PICS) during an episode of SAP had lower QoL scores overall, when compared with patients who had SAP only^26^.

### Aetiology of severe acute pancreatitis and quality of life

**Fig. 2:**
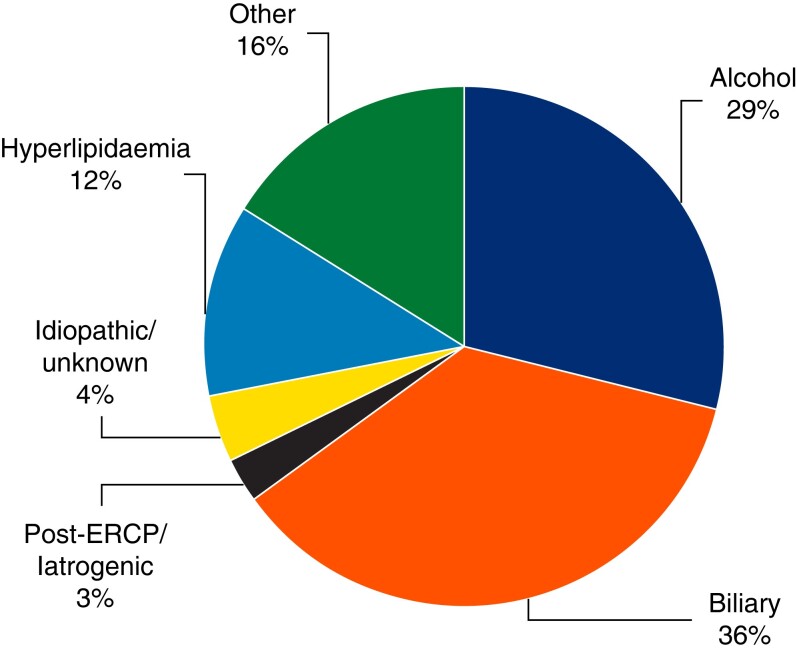
**Aetiology of severe acute pancreatitis in included patients from 14 cohort studies** .

The cause of SAP episodes and the QoL outcomes are detailed in *Tables 2* and *3*. All studies listed at least two causes of SAP, with the two most common being alcohol and biliary aetiology, followed by a significant proportion of hyperlipidaemia (*Fig. 2*).

The aetiology of SAP was analysed in 4 of 14 studies. Three of them revealed alcohol-induced pancreatitis patients as those having the lowest QoL scores^5,6,16^. One study demonstrated that alcohol-induced pancreatitis was linked to lower QoL scores, but also to increased calcification of the pancreas on imaging and exocrine insufficiency postnecrosectomy^6^.

### Quality of life and exocrine insufficiency

Ten studies investigated long-term exocrine insufficiency using either objective parameters (oral enzyme supplementation/reduced faecal elastase) or subjective parameters (reported symptoms of steatorrhea/diarrhoea/weight loss). A sum of 134 of 462 patients with SAP showed exocrine pancreatic insufficiency resulting in an estimated rate of 29 per cent. All studies with the highest reported rates of exocrine insufficiency, that is for more than 30 per cent of patients, also demonstrated a reduction in QoL but this was not statistically significant^18,22–24^.

### Quality of life and endocrine insufficiency

Eleven studies investigated the presence of endocrine insufficiency either as a new diagnosis of diabetes mellitus or impaired glucose tolerance^5,6,13,16–20,22–24^. A total of 185 of 588 patients (an estimated 31 per cent) showed endocrine insufficiency after an episode of SAP. The studies describing QoL as unchanged quoted rates of endocrine insufficiency ranging from 14 per cent to 79 per cent, and those concluding that QoL was reduced in at least one domain had endocrine insufficiency rates ranging from 5 per cent to 75 per cent (*Table 3*).

### Quality of life and chronic symptoms

Chronic disease symptoms were investigated and reported in six studies. Symptoms consisted of abdominal pain, distention, nausea and vomiting, change in bowel habit, neuropathy, and new-onset disability. There was no pattern observed between the rate of chronic symptoms and effect on QoL.

## Discussion

This systematic review summarizes how the variability of long-term effects are after an episode of SAP, ranging from a significantly reduced QoL to one comparable to healthy controls.

QoL after an SAP episode seemed to be affected by the severity grade of the episode. The idea that the higher the severity the greater the reduction in reported QoL is not novel, but confirmed what was previously described in the systematic review by Pezzilli *et al.*^35^.

An additional theme was the impact the aetiology of SAP has on QoL. In the case of an alcoholic aetiology, chronic implications were more frequent with the mental health component affected more than the physical^5,6,16,18^. It is not well established whether alcohol-related acute pancreatitis results in more severe disease, has longer lasting effects on the pancreatic gland, or results in a higher degree of exocrine insufficiency^36^. It is even harder to discern whether there is an element of chronic pancreatitis in this group of patients. Confounding social factors should be considered in alcohol-induced SAP including the concurrent use of other substances, mental health diagnoses, and social support available during the recovery period^32^.

It has been difficult to establish a clear relationship between pancreatic insufficiency and long-term QoL after SAP. This is mainly due to the heterogeneity of parameters chosen to characterize these functions across studies. A correlation between exocrine insufficiency and reduced QoL became apparent and even though not significant, it was more evident than one for endocrine insufficiency. Pancreatic function is the most investigated variable when assessing QoL and should therefore be assessed in a standardized way across future studies so that a clear relationship can be established.

**Fig. 3:**
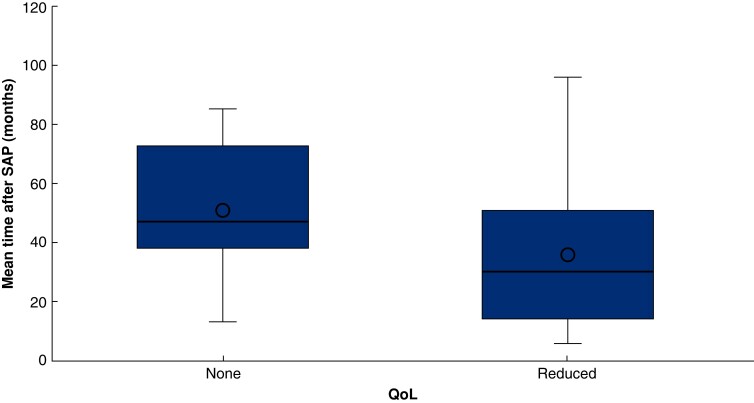
Mean follow-up time (and point at which QoL questionnaire completed) in months *versus* effect on QoL

QoL, quality of life; SAP, severe acute pancreatitis.

In addition, timing is an important variable in the assessment of QoL. QoL might be reduced in the immediate period after a SAP episode but return to normal as shown directly by one study as well as this review’s data synthesis^25^ (*Fig. 3*). This is most likely multifactorial, with a combination of biochemical changes, that is pancreatic function returning back to baseline after recovering from the acute insult, due to exocrine insufficiency improving in about 80 per cent of them^37^, and psychological changes, meaning that as patients learn to adapt to their condition they perceive their QoL to be better^18^. The timing of QoL assessment can therefore have an important impact on results^18^. As defined by Calman in 1984, QoL is the gap between a patient’s hopes and expectations and reality at any specific point in time^38^. As patients get used to their new reality, their perceived QoL improves.

Different grading systems were used in the assessment of QoL and were not disease specific. QoL is a multifaceted, dynamic parameter that is difficult to quantify and analyse. As previously described, a pitfall of QoL instruments is that they do not adequately consider the subjective experience of a disease and the effect of the intervention used^39^. QoL questionnaires are designed by healthcare professionals, hence can fail to include aspects that are important to patients’ QoL. A recent review of the tools used in the assessment of QoL after AP concluded that a disease-specific assessment is required and that the two most important determinants are acute clinical symptoms and the final nutritional status of a patient^31^.

The main limitation of the current review is the use of stringent inclusion criteria that resulted in a small number of eligible studies. Moreover, the heterogeneity of the studies included prevented conducting a meta-analysis.

Further research in the form of larger standardized studies is required. Identifying patients at risk of significant QoL reduction, during the initial SAP episode, will help direct treatment and can help improve their outcomes. A randomized controlled trial would be possible once standardized assessments have been developed to compare outcomes of different treatment modalities.

A disease-specific QoL assessment tool should be developed which incorporates subjective measures (social implications, perceived levels of health) as well as standardized objective measures (pancreatitis severity, exocrine and endocrine function, Intensive Treatment Unit (ITU) admission, length of stay (LOS), radiological/histological evidence of necrosis)^12,31^. The timing of such an assessment is important and should be taken into consideration perhaps by performing a bi-modal assessment: one after 12 months and one after 3–4 years. There should also be a standardized way of incorporating SAP aetiology, LOS, and surgical and medical intervention effects on QoL after SAP.

## Supplementary Material

zrad067_Supplementary_Data

## Data Availability

The data that support the findings of this study are available from the corresponding author, A.G., upon reasonable request.
