# Supplementary material for: Quality of life after severe acute pancreatitis: systematic review
Source: BJS Open. 2023 Aug 24;7(4):zrad067. doi: 10.1093/bjsopen/zrad067 (PMC10449419; doi:10.1093/bjsopen/zrad067)
Supplement: zrad067_Supplementary_Data [file zrad067_supplementary_data.docx]

**Quality of life after Severe Acute Pancreatitis: Systematic Review**

Andrea Giorga, Michael Hughes, Simon Parker, Andrew Smith, Alistair Young

Department of Pancreatic Surgery, St James’s University Hospital, Leeds Teaching Hospitals Trust, Leeds, UK, LS9 7TF

Corresponding author: Andrea Giorga, [agiorga@gmail.com](mailto:agiorga@gmail.com) / [andrea.giorga@nhs.net](mailto:andrea.giorga@nhs.net)

Address: Department of Pancreatic Surgery, St James’s University Hospital, Leeds Teaching Hospitals Trust, Leeds, UK, LS9 7TF

No Funding

Category: Review

No conflicts to declare

The authors confirm that the data supporting the findings of this study are available within the article [and/or] its supplementary materials.

**Supplementary Materials - Index**

| **Supplementary Appendixes** |  |
| --- | --- |
| Appendix S1 | *page 2* |

**Appendix S1:**

**Search Strategy**

Databases searched: MEDLINE, EMBASE, Scopus, PubMed

MESH terms:

- “Acute” AND
- “Severe” AND
- “Pancreatitis” AND
- “Quality of Life” OR “stress” OR “role emotional” OR “social behaviour” OR “mental health” OR “vitality” OR “general health” OR “pain” OR “role physical” OR “physical function” OR “activities of daily living” OR “fatigue” OR “adaptation” OR “well-being”

**Eligibility criteria**

Included

- Study Type: Prospective or Retrospective observational or interventional studies, Randomised Controlled Trials.
- Study Population:
  - Adult patients (>18 years old)
  - Patients with acute and not chronic pancreatitis. Where both types were included, data for ‘acute’ cases was extracted where possible.
  - Studies with assessment of severity of pancreatitis, if patients with different levels of pancreatitis severity were present, only the data on ‘Severe’ pancreatitis was extracted where possible.
- Study Outcome: Formal assessment of Quality of Life using validated questionnaires
- All aetiologies of acute pancreatitis, including alcohol, and analysed separately where possible.
- Any patients with confirmed necrotising pancreatitis were classified as having a severe episode of acute pancreatitis.

Excluded

- Study Type: Review Articles
- Year: Studies published before 2000
- Language: Any other than English and no translation available
- Studies in species other than Humans
- Studies for which full text articles were unavailable.
